# Genistein abrogates G2 arrest induced by curcumin in p53 deficient T47D cells

**DOI:** 10.1186/2008-2231-20-82

**Published:** 2012-11-30

**Authors:** Puji Astuti, Esti D Utami, Arsa W Nugrahani, Sismindari Sudjadi

**Affiliations:** 1 Pharmaceutical Biology Department, Faculty of Pharmacy, Universitas Gadjah Mada, Yogyakarta, Indonesia; 2 Pharmacy Department, Faculty of Medicinal and Health Sciences, Universitas Jenderal Soedirman, Purwokerto, Indonesia; 3 Sekolah Tinggi Ilmu Farmasi (STIFAR), Pharmacy Foundation, Semarang, Indonesia; 4 Pharmaceutical Chemistry Department, Faculty of Pharmacy, Universitas Gadjah Mada, Yogyakarta, Indonesia

**Keywords:** Cell cycle, p53, Curcumin, Genistein, G2 arrest

## Abstract

**Background:**

The high cost and low level of cancer survival urge the finding of new drugs having better mechanisms. There is a high trend of patients to be “back to nature” and use natural products as an alternative way to cure cancer. The fact is that some of available anticancer drugs are originated from plants, such as taxane, vincristine, vinblastine, pacitaxel. Curcumin (diferuloylmethane), a dietary pigment present in *Curcuma longa* rizhome is reported to induce cell cycle arrest in some cell lines. Other study reported that genistein isolated from *Glycine max* seed inhibited phosphorylation of cdk1, gene involved during G2/M transition and thus could function as G2 checkpoint abrogator. The inhibition of cdk1 phosphorylation is one of alternative strategy which could selectively kill cancer cells and potentially be combined with DNA damaging agent such as curcumin.

**Methods:**

T47D cell line was treated with different concentrations of curcumin and genistein, alone or in combination; added together or with interval time. Flow Cytometry and MTT assay were used to evaluate cell cycle distribution and viability, respectively. The presence of apoptotic cells was determined using acridine orange-ethidium bromide staining.

**Results:**

In this study curcumin induced G2 arrest on p53 deficient T47D cells at the concentration of 10 μM. Increasing concentration up to 30 μM increased the number of cell death. Whilst genistein alone at low concentration (≤10 μM) induced cell proliferation, addition of genistein (20 μM) 16 h after curcumin resulted in more cell death (89%), 34% higher than that administered at the same time (56%). The combination treatment resulted in apoptotic cell death. Combining curcumin with high dose of genistein (50 μM) induced necrotic cells.

**Conclusions:**

Genistein increased the death of curcumin treated T47D cells. Appropriate timing of administration and concentration of genistein determine the outcome of treatment and this method could potentially be developed as an alternative strategy for treatment of p53 defective cancer cells.

## Background

Most of chemotherapeutic drugs such as etoposide, cisplastin, doxorubicin and camptotechin induce DNA damage on cancer cells. These DNA (deoxy nucleid acid) damaging agents arrest or delay cell cycle progression, allowing time for DNA repair. This mechanism of repair functions to ensure that division only occur on cells carrying complete DNA, not mutated or damage. If the damage is beyond repair, the cells may permanently enter cellular senescence or undergo apoptosis [1,2]. The mechanism of cell cycle arrest is mediated by ATM (ataxia telangiectasia-mutated protein kinase) or ATR (ATM and Rad3-related protein kinase) through the activation of Ser/Thr kinases checkpoint kinase 1 (Chk1) and checkpoint kinase 2 (Chk2). The activation of Chk1 and Chk2 in turn modulates phosphorylation events such as phosphorylation of cdc25 phosphatases which normally activate cdk1 in G2/M boundary and result in cell cycle arrest at the G2/M or S phase [3]. Based on oncologic point of view, this mechanism of repair is beneficial to normal cells in that they have mechanism to reduce toxic effect of the chemotherapeutic agents. However, this system of protection limits the efficacy of chemotherapy on cancer cells.

In responds to DNA damaging agents, cell cycle arrest on G1 phase depends on tumor suppressor protein p53. Normal cells carrying wild type p53 are able to arrest at G1, S and G2 phase, whilst cells having defect on p53 gene, which occur on >50% of tumor, would progress through S phase and arrest at G2 phase [4-6]. Any agents which capable of abrogating cell cycle arrest at G2 phase would induce premature entry into mitosis, with cells still carry damaged DNA and resulting in apoptosis. Inducing mitotic catastrophe in G2 arrested cell can be used as a strategy to selectively kill tumor cell lacking functional p53, and at the same time provide opportunities for normal cells to survive.

Although conventional chemotherapy using DNA damaging agents resulting in tumor cell death, the deleterious side effects are well known. Furthermore, the high cost of treatment urges the finding of selective agents with more affordable price. Currently, there is trend of patients to use natural medicine as an alternative therapy against cancer. The fact that some of available anticancer drugs such as taxan, vincristine, vinblastine and pacitaxel are medicinal plant origin, provide great opportunities to effectively use natural compounds to treat cancer. A lot of studies have been conducted to examine the potential of natural compounds against cancer with one of example is curcumin.

Curcumin (diferuloylmethane) is dietary pigment and presence as major compound in *Curcuma longa rizhome.* This compound has a potential to be developed as anticancer agent and was in phase II clinical trials [7,8]. Curcumin was reported to be cytotoxic and inactive in normal and primary cells. In mouse embryonic fibroblast line C3H/10 T1/2, rat embryonic fibroblasts, and human foreskin fibroblast, curcumin did not induce cell death, whereas in cancer cells it stimulated cell death through mechanism of apoptosis [8-12]. Curcumin also induced cell cycle arrest in colorectal tumor line HCT116, medulloblastoma and human acute promyelocytic leukemia HL-60 [12-14]. The ability to induce cell death increased with addition of piperine, the major compound of *Piper nigrum* L) which reportedly increase the bioavailability of curcumin [14-17].

The activity of curcumin as anticancer agent can be increased by combination with compounds having effect as G2 checkpoint abrogator. Flavonoid is natural polyphenolic compounds potential to be developed as anticancer agents [18,19]. Isoflavonoid genistein was found to be active in pancreatic cells by modulating cell cycle and inhibition of angiogenesis [20,21]. Following administration of irinotecan, this compound inhibited phosphorylation of cdk1 mediated by wee1 kinase, a negative regulator of cdk1 kinase activity [22]. Inhibition of cdk1 phosphorylation could be a potential strategy to abrogate G2 checkpoint activation [23].

## Methods

### Materials

Genistein was obtained from Sigma, curcumin was kindly given by Dr. Hilda Ismail, 86% purity by HPLC (High Pressure Liquid Chromatography). They were dissolved in ethanol absolute, divided into aliquots, and stored frozen at −20°C.

### Cell lines and culture conditions

T47D (Human ductal breast epithelial tumor cell line) was cultured in RPMI 1640 media supplemented with 10% Fetal Bovine Serum (Gibco) 2% Penicillin - Streptomycin (Gibco), dan 0.5% Fungizon (Gibco), 2% Sodium bicarbonate (Gibco) and HEPES (4-(2-hydroxyethyl)-1-piperazineethanesulfonic acid) (Invitrogen). Cell lines were maintained at 37°C in a humidified incubator containing 5% CO_2_.

### Cell cycle analysis

Treated cells were harvested using trypsine and washed three times with 1×PBS. Cell pellets were resuspended with 500 μl staining solution (40 μg/ml propidium iodide (Sigma) and 500μg/ml RNase A (Sigma), covered with aluminium foil and incubated at 37°C for 30 minutes. Cells were analysed using FACS (Fluorescence Activated Cell Sorting) Calibur (BD).

### Viability assay

A hundred μl of media containing 5x10^3^ cells was added to 96-well plate and incubated for 48 hours until 70% – 80% confluent. Curcumin was added alone or in combination with genistein, added together or in interval time. Cells were incubated at 37°C in CO_2_ incubator. Following the treatment, cells were gently washed with 1X PBS (Phosphate Buffer Saline), and 100 μl of MTT (3-(4,5-Dimethylthiazol-2-yl)-2,5-diphenyltetrazolium bromide) 0.5 mg/ml was added to the well. The cells were incubated for 4 hours at 37°C and the reaction was stopped by adding 100 μl SDS (sodium dodecyl sulfate) 10%. The plates were incubated overnight and read in microplate reader (Bio-Rad) at 595 nm.

### Apoptotic assay

T47D cells were plated onto 6 well plate containing coverslip and treated with curcumin, genistein or in combination as indicated. Following the treatment, the cells on coverslip were treated with ethidium bromide-acridine orange and the cells were analysed under fluorescence microscope (Carl Zeiss).

## Results

### Curcumin induced G2 arrest in T47D cells

**Figure 1:**
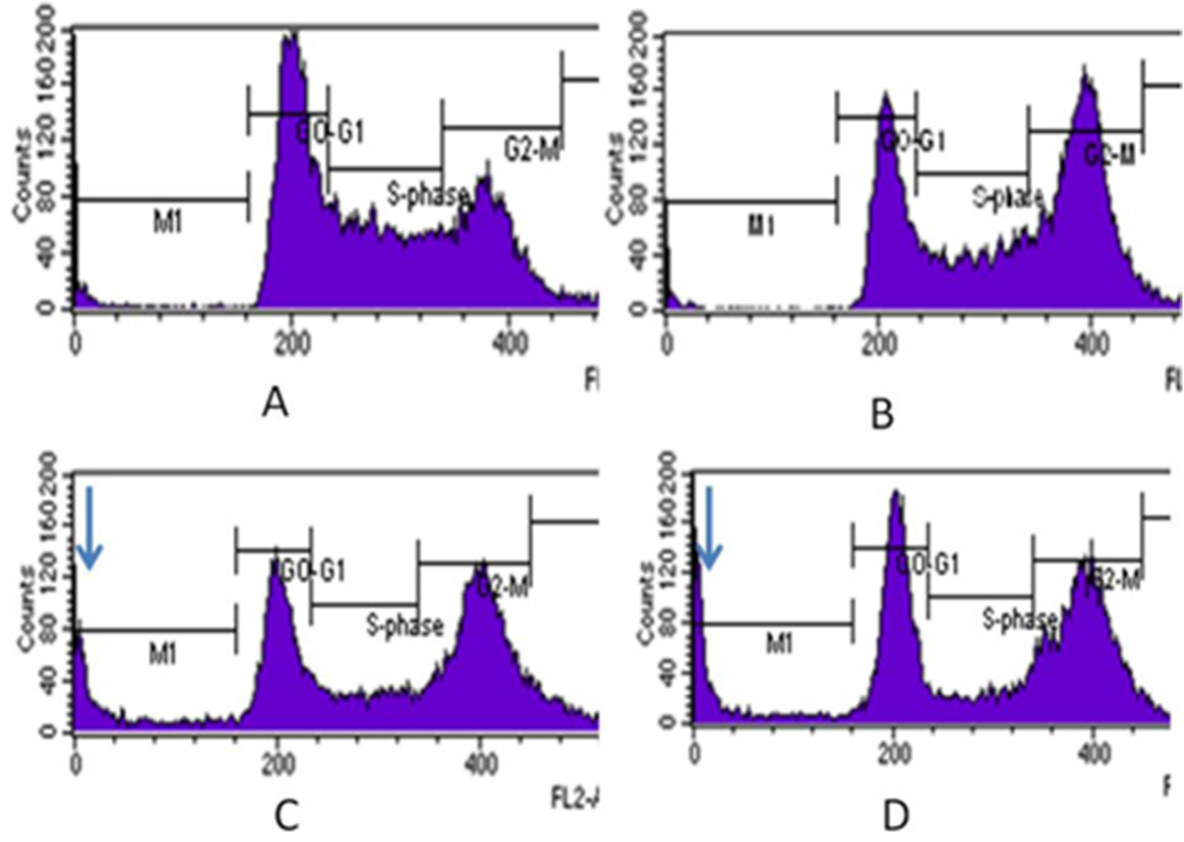
**Cell cycle analysis of T47D cell using flowcytometry after treatment with different concentration of curcumin.** T47D cells were plated onto 6 well plate and treated overnight with (**A**) control untreated cells, (**B**) 10 μM curcumin, (**C**), 20 μM curcumin and (**D**) 30 μM curcumin. Arrow indicated the presence of sub G1 population.

To study the mechanism of action of curcumin in vitro, we have tested the effect of increasing concentration of curcumin in inducing G2 arrest in T47D cells. The cells were treated at different concentration of curcumin and incubated overnight. As shown in Table 1 and Figure 1, curcumin at 10 μM induced G2 arrest in T47D cells. The cells showed a strong block in the G2/M phase of the cell cycle (reaching 44% compared with 25% in the untreated controls). Increasing the concentration up to 30 μM did not significantly increase G2/M population. Instead, it appeared a marked sub-G1 peak (from 2% in control cells to 12% at 20 μM and 14% at 30 μM of treated cells), because of the presence of dead cells. Adding curcumin at low as 5 μM did not induce cell cycle arrest (data not shown). These data demonstrate that curcumin at 10 μM arrest T47D cells at G2/M with little cytotoxicity.

**Table 1:** Cell cycle distribution of T47D cells after treatment of different concentration of curcumin overnight

**Treatment**	**Sub G1 (%)**	**G1 (%)**	**S (%)**	**G2 (%)**
Control	2.32	37.23	27.88	25.34
10 μM curcumin	1.26	22.74	19.30	44.13
20 μM curcumin	12.78	22.13	13.79	37.43
30 μM curcumin	14.28	28.38	11.39	39.57

### Genistein abrogated G2 arrest induced by curcumin

**Figure 2:**
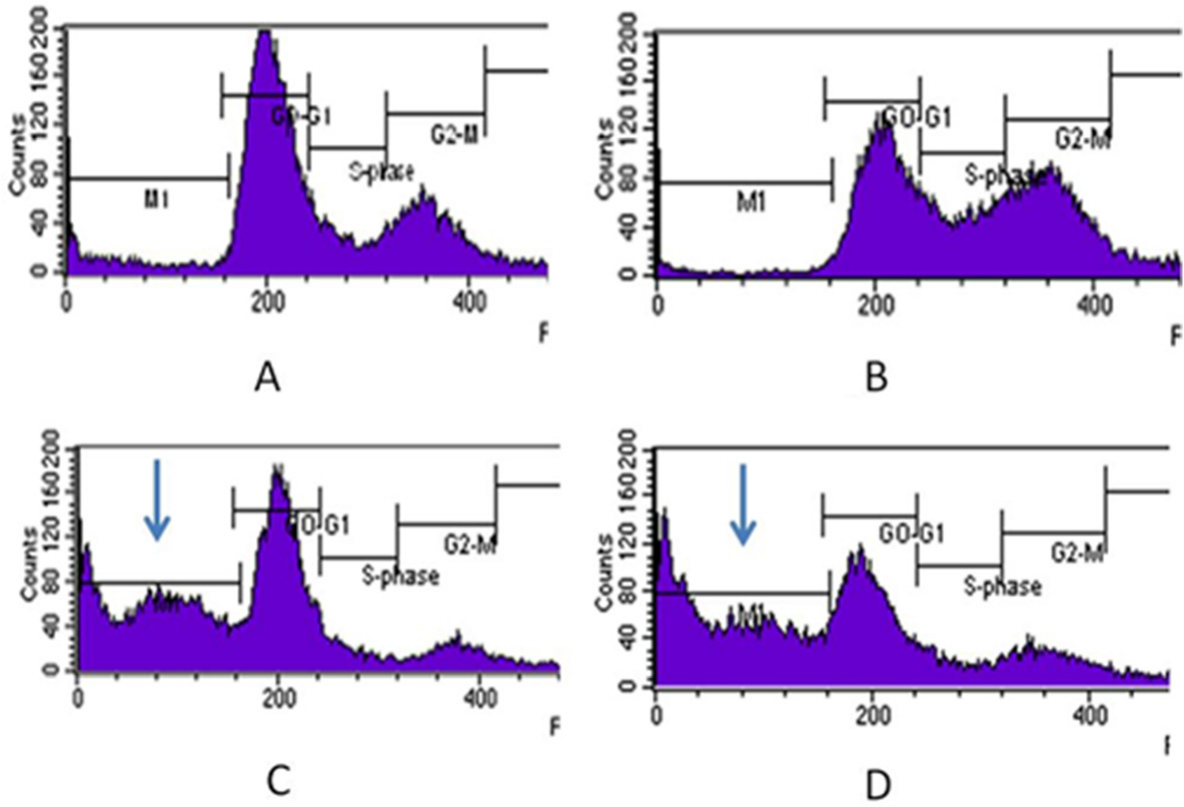
**Cell cycle analysis of T47D cell using flowcytometry after treatment with curcumin and genistein.** T47D cells were plated onto 6 well plate and treated with (**A**) control untreated cells, (**B**) 10 μM curcumin, (**C**), 10 μM curcumin overnight followed by 20 μM genistein and (**D**) 10 μM curcumin overnight followed by 50 μM genistein. Arrow indicated the presence of subG1 population.

In an attempt to discover natural compounds that disrupt G2 checkpoint in cancer cells, we used genistein, an inhibitor of cdk1 phosphorylation [22]. We examine the ability of different concentration of genistein in modulating G2 arrest induced by curcumin. Firstly, we activated the G2 checkpoint by adding curcumin at the concentration of 10 μM overnight. Sixteen hours after DNA damage, the cells were treated with different concentration of genistein for another 24 hours. The cells were harvested and the cell cycle distribution was analysed. Indeed, as shown in Table 2 and Figure 2, genistein at concentration of 20 μM abrogated the G2/M block induced by curcumin, decreasing G2/M population from 28% in curcumin only treated cells to 6% in combination with 20 μM genistein. Increasing concentration of genistein up to 50 μM did not significantly reduce the shift of G2/M population. The G2/M population only decreased to 10% in combination with 50 μM genistein. Combination of curcumin with genistein increased the subG1 population which represents dead cells, from 2% to 44% (20 μM genistein) and 47% (50 μM genistein).

**Table 2:** Cell cycle distribution of T47D cells after treatment of 10 μM curcumin overnight followed by 20 μM genistein or 50 μM genistein

**Treatment**	**Sub G1 (%)**	**G1 (%)**	**S (%)**	**G2 (%)**
Control	8.51	48.85	11.13	18.52
10 μM curcumin	2.71	31.12	18.86	28.22
10 μM curcumin + 20 μM genistein	44.25	39.66	5.72	6.44
10 μM curcumin + 50 μM genistein	47.47	29.17	6.76	10.14

### Addition of genistein following G2 arrest induced by curcumin resulted in more cell death

**Figure 3:**
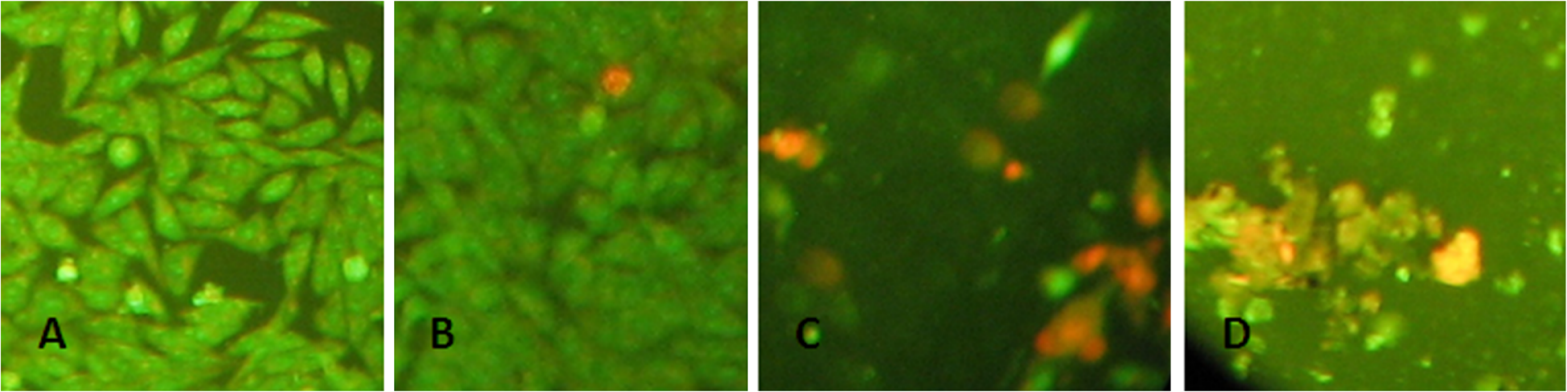
**Morphology of cells treated with curcumin, genistein or in combination.** T47D cells were plated onto 6 well plate and treated with (**A**) control cells, (**B**) 10 μM curcumine only, (**C**) curcumin (10 μM) was added overnight followed by treatment with genistein (20 μM), (**D**) curcumin (10 μM) was added overnight followed by treatment with genistein (50 μM).

To check the effect of combination of genistein with curcumin on cell viability, we treated the cells with curcumin, genistein and in combination with various interval times by means of MTT assay. The experiments were performed in three replicates. Previously we found that curcumin at concentration of 10 μM induced G2/M arrest with little toxicity. In this experiment we confirmed the finding that treatment curcumin at given concentration retained the viability of cells of 83.66%. An attempt to increase the sensitivity of this compound in inducing cell death is conducted by addition of genistein. Table 3 shows that genistein alone at low concentration of 5 and 10 μM induced cell proliferation as compared to control, reaching 191.80% and 171.22% respectively. Increasing concentration to 20 μM maintained the viability of 90.41%, whereas high concentration of 50 μM induced 61.28% cell death. In this study we found that genistein (20 μM) which was added 16 hours after 10 μM curcumin resulted in more cell death, from 83.66% viability in curcumin only treated cells to only 10.88% in combination. Adding higher concentration of genistein (50 μM) only slightly increased the percentage of cell death (8.48%). Interestingly, in this study we found that adding genistein following DNA damage induced by curcumin produced more cell death (89.12%) compared to adding at the same time (56.51%). The effect of inducing cell death by combination of curcumin and genistein were confirmed by observing the morphology of the cells after treatment with curcumin or in combination with genistein using ethidium bromide and acridine – orange double staining under fluoresence microscope. The presence of apoptotic cells were shown as orange to red population as compared to healthy green cell population [24]. As shown in Figure 3, only few dead cells were observed in curcumin only treated cells. However, combining curcumin with genistein resulted in high accumulation of apoptotic cells. Combination with high concentration of genistein induced the presence of necrotic cells, as appear as a form of debris (Figure 3).

**Table 3:** Persentage of cell viability following the administration of curcumin, genistein and combination (n =3)

**No**	**Treatment**	**% viability ± SE**
1	Curcumin 10 μM	83.66 ± 8.24
2	Genistein 5 μM	191.80 ± 8.94
3	Genistein 10 μM	171.22 ± 6.63
4	Genistein 20 μM	90.41 ± 2.13
5	Genistein 50 μM	38.72 ±3.99
6	Curcumin 10 μM + Genistein 20 μM*	10.88 ± 1.85
7	Curcumin 10 μM + Genistein 50 μM*	2.40 ± 0.59
8	Curcumin 10 μM + Genistein 20 μM**	43.49 ± 4.90
9	Curcumin 10 μM + Genistein 50 μM**	15.83 ± 0.88

** Curcumin and genistein were added at the same time. * Genistein was added 16 hours after the addition of curcumin. These data represent minimal two independent experiments.

## Discussion

Conventional chemotherapeutics have been widely applied in treatment against various type of cancer. The effectiveness of these therapies depends on their ability to kill proliferating cancer cells by damaging their DNA and inducing apoptosis. The cell responds to DNA damage to induce cell cycle arrest at any stage, ensuring the damage is repaired prior to enter subsequent phase of cell cycle [25]. This mechanism of control is function to maintain genetic integrity, with the failure of repair resulted in mutations and eventually cell death. However, these agents convey some drawbacks in which they developed resistance to DNA damage-induced cell killing and they are also toxic to actively proliferating normal cells.

One of the characteristics of tumor growth is uncontrolled cell proliferation as a result of loss in normal cell cycle control. Currently there is an increasing of interest to target cell cycle in effort to find targeted anticancer therapies [26-29]. In fact >50% of human cancer have loss in the tumor suppressor gene p53. This gene is an essential component for apoptosis induction in response to DNA damage and also a component of cell cycle checkpoint. The loss of p53 resulted in cell reliance on G2 phase checkpoint in response to DNA damage inducing agent which easily can be bypassed by adding G2 phase checkpoint inhibitors to induce aberrant mitosis and eventually increased cell death [30,31].

In Indonesia, there is a high trend of using herbal medicine in promoting health with some of the herbal materials commonly used are curcuma rizhome (*Curcuma longa* L) and soy bean (*Glycin max*) which contain bioactive compound of curcumin and genistein, respectively. The bioactivities of these natural products have been widely reported [13,15,21,32]. In this study we tested the combined effects of genistein with curcumin in inhibiting the growth of cancer cells. As was seen in T47D cells, curcumin induces G2 phase arrest, similar as reported by others [12,13] and genistein abrogated the G2 checkpoint controls induced by curcumin. The combination of the two agents reduced cell viability up to seven fold, in comparison of adding the agent alone. Since many of cancers have defective of checkpoint control due to the loss of p53 gene, further deregulation of this mechanism of control can result in increased apoptotic cell death.

In this study we demonstrated the success of applying strategies to deliver a more cancer-specific cytotoxic treatment by synthetic lethality targeting p53 defective cancer cells. These synthetic lethality strategies are currently being used to search new drugs or targets within specific tumor types such as inhibition of Chk1 in p53 mutant cancers to achieve better outcomes [33,34]. Using this strategy, reduced side effects and better outcomes could be achieved since doses of chemotherapeutic agents can be reduced at least twofold [35-37]. In this study we showed the selectivity of synthetic lethality strategy by targeting the loss of function of p53 and application of DNA damage inducing agent. In T47D, p53 deficient cells, survival of the cells following DNA damage are dependent on activation of the Chk1 pathway to induce G2 cell cycle arrest and DNA repair. Disruption of this Chk1-dependent pathway by genistein can selectively sensitize cells to exit G2 phase before the damage are repaired and eventually the cells undergo apoptosis. In this study we demonstrated that adding combination of curcumin followed by genistein on T47D cells showed greater cell death, indicating the importance of appropriate timing of administration in inducing cell death. Similar study was reported by Tse et al. [38], when combining topoisomerase I posion and checkpoint inhibitor 7-hydroxystaurosporine in human colon cancer. Sequential treatment of SN-38 followed by UC-01, a Chk1 inhibitor, resulted in enhanced apoptosis in p53 mutant cells, and this was proven to be effective in p53 mutant but to lesser degree in p53 intact cells.

Collectively, these studies provide a new insight in using naturally derived agents to treat cancer with selective methods to achieve better outcomes. This approach is based on understanding that in the synthetic lethal strategy targeting p53 defective cells, the loss of stress response mechanism can reduce cell viability. This response of mechanisms, when combined with the loss of compensatory systems can generate complete loss of cell viability [33,39].

## Conclusions

In this study we demonstrated that genistein increased the death of curcumin treated T47D cells. We showed that the inhibition of checkpoint pathway is generated by genistein and the selectivity is achieved by the loss of p53 function in T47D cells and application of curcumin. Since the main active compounds used in this study are natural product origin, it is expected that this study could support the rational of using traditional medicines for maintaining health. Appropriate use of these natural products could help cancer patients in using alternative therapies with scientific evidence based medicines.

## Competing interest

The authors declare that there is no conflict of interest related to this publication.

## Authors’ contributions

PA contributes to the concept, design, data acquisition, analysis and interpretation; preparation of the manuscript and approval of final article. EDU contributes to data acquisition and analysis, preparation of the manuscript and approval of final article. Part of the data was collected for her master thesis. AWN participated in data acquisition and analysis and helped to draft the manuscript. SS participated in the design and coordination, preparation of the manuscript. All authors read and approved the final manuscript.
